# Quantification of acute myocardial injury by ShMOLLI T1-Mapping, T2-weighted and late gadolinium imaging in patients presenting with chest pain, positive troponins and non-obstructive coronary arteries

**DOI:** 10.1186/1532-429X-13-S1-P16

**Published:** 2011-02-02

**Authors:** Vanessa M Ferreira, Stefan K Piechnik, Erica Dall'Armellina, Theodoros D Karamitsos, Jane M Francis, Matthias G Friedrich, Matthew D Robson, Stefan Neubauer

**Affiliations:** 1 University of Oxford, Oxford, UK; 2 Stephenson Cardiovascular MR Centre, Libin Cardiovascular Institute of Alberta, Calgary, AB, Canada

## Objective

To assess the ability of ShMOLLI T1-mapping in detecting acute myocardial injury in patients with chest pain, positive troponins and non-obstructive coronary arteries.

## Background

To diagnose acute myocardial injury of varying etiologies, cardiovascular magnetic resonance (CMR) techniques must be sensitive to global and focal changes. ShMOLLI T1-mapping permits quantitative myocardial characterization without contrast agents or reference regions. We compare ShMOLLI T1-mapping against traditional CMR imaging modalities.

## Methods

16 patients (8 men, mean age 51±16 years) with acute chest pain, Troponin I >1.0 ug/L and non-obstructive coronary arteries underwent 1.5T CMR imaging within 7 days of presentation (median 3 days). CMR protocol included Shortened Modified Look-Locker inversion recovery (ShMOLLI) for T1-mapping, T2-STIR and late gadolinium enhancement (LGE) imaging. Regional wall motion was assessed with SSFP cines. Volumetric fractions of injured myocardium were quantified by (1) T2 signal intensity (SI) of myocardium:skeletal muscle >1.9:1; (2) T1>110% of normal myocardial T1; and (3) LGE SI>2SD of remote myocardium SI.

## Results

**Figure 1:**
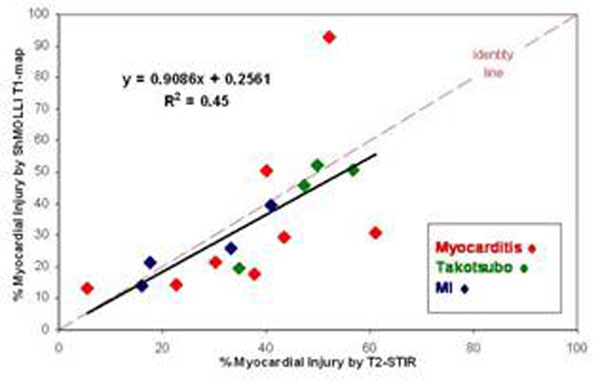
Myocardial injury by ShMolli T-Mapping and T2-STIR

8 cases of myocarditis, 4 cases of Takotsubo cardiomyopathy and 4 cases of myocardial infarction were diagnosed based on CMR findings. All demonstrated abnormalities by at least 2 out of the 3 sequences (*Table*1). All but one (focal myocarditis) demonstrated global/focal increase in T2 SI. All demonstrated global/focal increase in T1 values. Except for patients with Takotsubo cardiomyopathy, all demonstrated LGE. Volumetric fractions of injury by T2-STIR and T1-mapping overlapped but did not necessarily co-localize topographically; there was moderate correlation between the two methods (R^2^=0.45) (*Fig.*1).

**Table 1:** Volumetric fraction of acutely injured myocardium

Patient Groups	By T2 STIR (>1.9:1)	By T1-mapping (T1_Normal_ x 110%)	By LGE (>2SD)
Takotsubo (n=4)	47±9%	42±15%	10±5%
Myocarditis (n=8)	37±17%	34±27%	18±6%
Myocardial infarct (n=4)	27±12%	25±11%	25±10%
All	37±16%	34±21%	18±8%

Patients with Takotsubo cardiomyopathy (mean EF=54±7%) had the highest average T1 values (1028±34ms vs. 968±76ms in normal controls; p<0.025*). Except for one with global myocarditis, patients with myocarditis and MI were without significant LVEF impairment with focal injury. Accordingly, average T1 values in these subgroups were not statistically different from normal.

**Figure 2:**
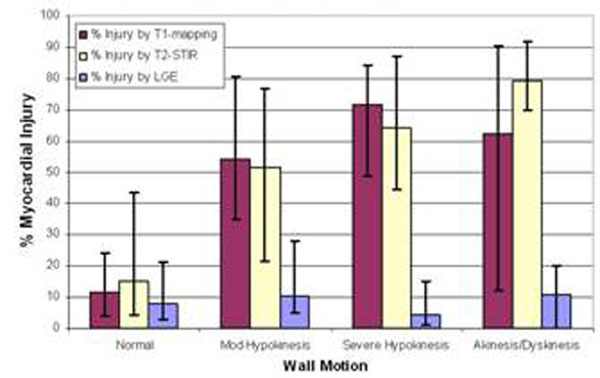
%Myocardial injury by T1-Mapping, T2-STIR and LGE

On a regional level, compared to segments with normal wall motion, segments with abnormal wall motion had significantly increased T1 values (958±60 ms vs.1068±76 ms, respectively; p<0.001*) (*Fig.*2), larger fractions of injury by T1-mapping (median 12% vs. 62%; p<0.001*), T2-STIR (15% vs. 62%; p<0.001*) and LGE (8% vs 10%; p<0.04*). LVEF correlated best with T1-mapping derived fractions of injured myocardium (R^2^=0.59; T2-STIR R^2^=0.18, LGE R^2^=0.1).

## Conclusion

ShMOLLI cardiac T1-mapping objectively detects acute global and focal myocardial injury of non-ischaemic and ischaemic etiologies. It is a promising method for acute CMR imaging with potential to offer adjunctive information in the characterization of acute myocardial injury.

